# Effects of Freeze-Drying Processes on the Acoustic Absorption Performance of Sustainable Cellulose Nanocrystal Aerogels

**DOI:** 10.3390/gels10020141

**Published:** 2024-02-12

**Authors:** Ju-Qi Ruan, Kai-Yue Xie, Jun-Nan Wan, Qing-Yuan Chen, Xiaoqing Zuo, Xiaodong Li, Xiaodong Wu, Chunlong Fei, Shanshan Yao

**Affiliations:** 1 School of Physics Science and Technology, Kunming University, Kunming 650214, China; 2 School of Materials Science and Engineering, Jiangsu University, Zhenjiang 212013, China; 3 Faculty of Materials Science and Engineering, Kunming University of Science and Technology, Kunming 650093, China; 4 College of Materials Science and Engineering, Nanjing Tech University, Nanjing 210009, China; 5 School of Microelectronics, Xidian University, Xi’an 710126, China

**Keywords:** cellulose aerogel, conventional freezing, unidirectional freezing, acoustic absorption, multifunction

## Abstract

Cellulose aerogels have great prospects for noise reduction applications due to their sustainable value and superior 3D interconnected porous structures. The drying principle is a crucial factor in the preparation process for developing high-performance aerogels, particularly with respect to achieving high acoustic absorption properties. In this study, multifunctional cellulose nanocrystal (CNC) aerogels were conveniently prepared using two distinct freeze-drying principles: refrigerator conventional freezing (RCF) and liquid nitrogen unidirectional freezing (LnUF). The results indicate that the rapid RCF process resulted in a denser CNC aerogel structure with disordered larger pores, causing a stronger compressive performance (Young’s modulus of 40 kPa). On the contrary, the LnUF process constructed ordered structures of CNC aerogels with a lower bulk density (0.03 g/cm^3^) and smaller apertures, resulting in better thermal stability, higher diffuse reflection across visible light, and especially increased acoustic absorption performance at low–mid frequencies (600–3000 Hz). Moreover, the dissipation mechanism of sound energy in the fabricated CNC aerogels is predicted by a designed porous media model. This work not only paves the way for optimizing the performance of aerogels through structure control, but also provides a new perspective for developing sustainable and efficient acoustic absorptive materials for a wide range of applications.

## 1. Introduction

Noise has emerged as a significant environmental concern in the 21st century, necessitating urgent measures to alleviate the distress caused by noise in various scenarios, including high-end equipment manufacturing, construction, transportation, and daily life. The adopting of sound-absorbing materials is the most effective way to eliminate noise. Based on the principle of dissipating sound energy, sound-absorbing materials can mainly be divided into four categories: porous material [1], (micro-)perforated plate, metamaterial [2], and a hybrid type [3]. Among these, porous materials offer advantages such as a larger absorption bandwidth, cost-effectiveness, and space efficiency, making them highly promising for practical applications [4,5,6]. However, given the inadequacy of existing porous sound absorption materials in terms of sustainability, a green and highly efficient acoustic absorptive material is still actively being explored.

Aerogels are three-dimensional micro/nanoporous solid materials with air as the dispersion medium which have been widely applied in various sectors including thermal insulation [7], catalysts [8], sensors [9], drug delivery [10], energy storage [11], etc. Due to their unique physical properties, such as ultra-high porosity, ultra-low density, and sound propagation speed, aerogels are also excellent acoustic absorption materials to efficiently suppress noise [1,12,13]. Cellulose aerogels, as a new type of bio-based porous material, not only inherit the superior porous structures of traditional aerogels, but also possess extremely high sustainable value, showing good prospect in the field of noise reduction [14,15,16].

The absorption of sound energy by porous materials occurs through their interconnected pores due to viscous, thermal, and inertial effects caused by the interaction of air molecules at the interfaces of the gas and the solid phases [15]. Hence, the design and regulation of the pore structures of cellulose aerogels play an important role in optimizing their acoustic absorption performance. It has been reported that the porous characteristics of cellulose aerogels can be effectively regulated through cellulose content [17,18,19], amounts and types of crosslinker or aging agent [16,19,20], intensity of hydrogen bonds [21], drying processes, etc. In particular, controlling their physical properties (e.g., porosity, density, and aperture) with the aid of different drying principles appears to be a convenient approach to efficiently regulate porosity features. Compared to supercritical [22,23] or atmospheric [24,25] drying, the freeze-casting technique [26] is an optimal strategy for producing cellulose aerogels on a large scale. It involves freezing a liquid suspension and subliming the solvent thereafter under ultra-low pressure. During the freezing process, the suspended cellulose fibers are organized by rejection from the growing ice crystal front to the intervening space, resulting in a porous structure after sublimation [27]. Thus, the freezing rate (affected by the freezing temperature) is an important factor in regulating the porous structure, especially the pore size, of cellulose aerogels by controlling the growth speed of ice crystals. In a typical study, Mei et al. [28] investigated the effect of freezing rate on the structure and properties of a lignocellulose aerogel. The results indicated that with an increase in freezing temperature from −196 °C to −30 °C and then to −18 °C, the fabricated aerogel possesses increasing apertures of ~15 μm, ~50 μm, and ~150 μm with higher water absorption capacity. Xu et al. [29] prepared a kind of cellulose nanofiber composite aerogel through freeze-drying by using liquid nitrogen (−196 °C) and a refrigerator (−40 °C) as the cooling source. Due to the refrigerator freezing method having a much slower freezing rate and thus producing larger ice crystals, the obtained aerogel exhibited a larger pore size of nearly 70 µm compared to the aerogel frozen by liquid nitrogen (nearly 10 µm). Meanwhile, numerous studies have confirmed that ice crystals nucleate quickly and grow rapidly to form fine structures at relatively low temperatures, while at higher temperatures, ice crystal nucleation and growth rates slow down, leading to an adequate growth of ice crystals and the formation of porous structures with larger pore sizes [27,30,31]. Temperature gradient (controlled by directional freezing) is another key factor regulating the three-dimensional porous structures of cellulose aerogels. Different from conventional freezing, directional drying is particularly capable of designing anisotropic pore structures by guiding the orderly growth of ice crystals. Unidirectional freezing, as a representative method, can generate a temperature difference from the bottom to the top, causing the ice crystal to grow along the temperature gradient, resulting in a lamellar structure along the growth direction of ice crystals [30,32]. For instance, xyloglucan/cellulose [32] and silk fibroin–cellulose [33] nanocrystal aerogels fabricated by unidirectional freeze-casing both exhibit an oriented lamellar structure with ordered pore channels, showing superior compressive performance along the freezing direction. In recent years, a bidirectional freezing technique has been developed to assemble small building blocks into large-scale aligned layers, forming a hierarchical porous structure by controlling the temperature gradient both from horizontal and vertical directions [34,35]. Liu et al. [36] prepared a multifunctional bianisotropic polyimide/bacterial cellulose aerogel in which bridge-like structures were successfully constructed between the layered skeletons via bidirectional freezing technology, resulting in excellent resilience performance. Additionally, other freezing principles, including cyclic freezing–thawing [30,37], spray freezing [38], dual ice-templating assembly [39], substrate wettability regulation [30], etc., were also reported to construct functional porous structures for cellulose aerogels (e.g., uniform or mesoporous structures).

The aforementioned studies successfully designed and regulated the porous structures of cellulose aerogels based on various drying principles, showing excellent performance in aspects of pollutant adsorption [25,29,34,35,39], infrared shielding [39], thermal insulation and management [25,30,36,38,39], and soft-tissue engineering applications [33]. However, only a few studies focused on acoustic absorption behaviors [40,41,42,43]. The acoustic absorption of a porous material is closely related to its pore structure features [44]. Therefore, it is of great significance to investigate and optimize the acoustic absorption properties of cellulose aerogels by adjusting their pore structures through specific drying mechanisms. In this study, we investigated the construction mechanism of porous structures for cellulose nanocrystal (CNC) aerogels depending on the growth pattern of ice crystals via two typical freezing methods, i.e., refrigerator conventional freezing (RCF) and liquid nitrogen unidirectional freezing (LnUF). The acoustic absorption properties influenced by the resulting porous structures of fabricated CNC aerogels were studied both using an experimental method and via a simple three-parameter analysis model derived from the Johnson–Champoux–Allard–Lafarge (JCAL) theory. The results may provide valuable guidance for efficiently producing versatile aerogels with predictable acoustic absorption performance by means of appropriate drying techniques.

## 2. Results and Discussion

### 2.1. Characteristics of the Porous Structures of CNC Aerogels

**Figure 1:**
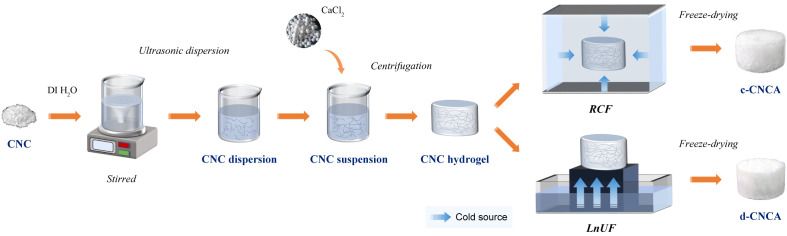
Schematic illustration of the preparation of cellulose nanocrystal (CNC) aerogels via two different freeze-drying principles. RCF represents conventional refrigerator freezing and LnUF represents liquid nitrogen unidirectional freezing.

CNC aerogels were fabricated through a simple dispersion and aggregation process by selecting CaCl_2_ as the green crosslinker. Afterward, they were dried by two different freeze-drying pathways (RCF and LnUF), as shown in Figure 1. The specific preparation process can be found in Section 4 (Materials and Methods).

**Figure 2:**
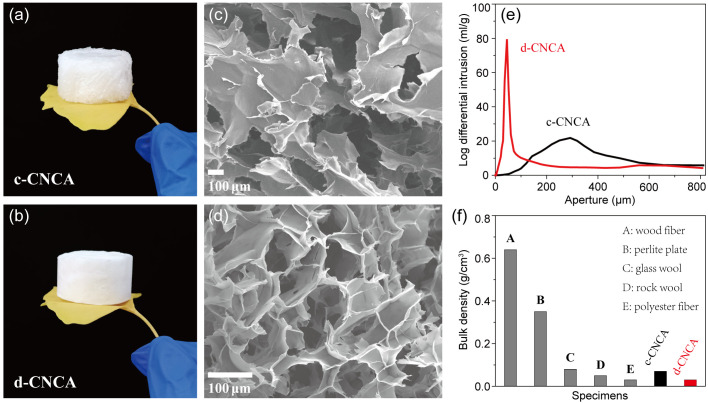
Optical photographs of c-CNCA (**a**) and d-CNCA (**b**). SEM images of c-CNCA (**c**) and d-CNCA (**d**). (**e**) Pore size distribution pattern of c-CNCA and d-CNCA. (**f**) Comparison of bulk density between c-CNCA and d-CNCA as well as several commonly used porous sound-absorbing materials.

Figure 2a,b show an optical photograph of ultra-light c-CNCA and d-CNCA, respectively. The sublimation process during freeze-drying effectively prevents the destruction of the porous structure caused by capillary force, resulting in highly complete CNC aerogels without significant contraction [45]. From the optical photographs, it can be seen that c-CNCA possesses a rough surface compared to that of d-CNCA. Figure 2c,d show SEM images of c-CNCA and d-CNCA. Obviously, c-CNCA presents a disordered structure with an irregular arrangement of larger pores, while d-CNCA exhibits an ordered structure with neatly arranged pores and smaller apertures. The structural differences between c-CNCA and d-CNCA stem from the growth pattern of ice crystals during the freezing process. In the RCF process for c-CNCA, the relatively high freezing temperature (−20 °C) leads to a slower nucleation and growth rate of ice crystals. Adequately grown ice crystals reject the CNC skeleton in any direction when subjected to an omnidirectional cold source, forming a disordered CNC network with large pores between the boundaries of neighboring ice crystals [46]. On the other hand, during the LnUF process, ice crystals nucleate and grow quickly along the direction of the temperature gradient driven by a unidirectional ultra-low-temperature cold source, resulting in a fine and ordered porous structure in d-CNCA. The differences in the porous structures of c-CNCA and d-CNCA are further quantitatively confirmed in Figure 2e and Table 1. The pore size distribution of d-CNCA is mainly concentrated in a smaller pore range, from 30 to 60 μm, hence resulting in smaller feature apertures as well as a lower structural permeability than that of c-CNCA (Table 1). Additionally, the as-prepared CNC aerogels exhibit a high porosity above 90% (Table 1) and an ultra-low bulk density (0.07 g/cm^3^ of c-CNCA and 0.03 g/cm^3^ of d-CNCA). The porosity of d-CNCA is relatively higher than that of the cellulose-based aerogels reported in the literature [20,41], and its density is also lower compared to several reported cellulose aerogels [20,36,41,43] as well as commonly used porous sound-absorbing materials (Figure 2f). The significant distinction between the porous structures of c-CNCA and d-CNCA will inevitably lead to different physical and chemical properties, especially in terms of their acoustic absorption performance.

### 2.2. Chemical Structures of CNC Aerogels

**Figure 3:**
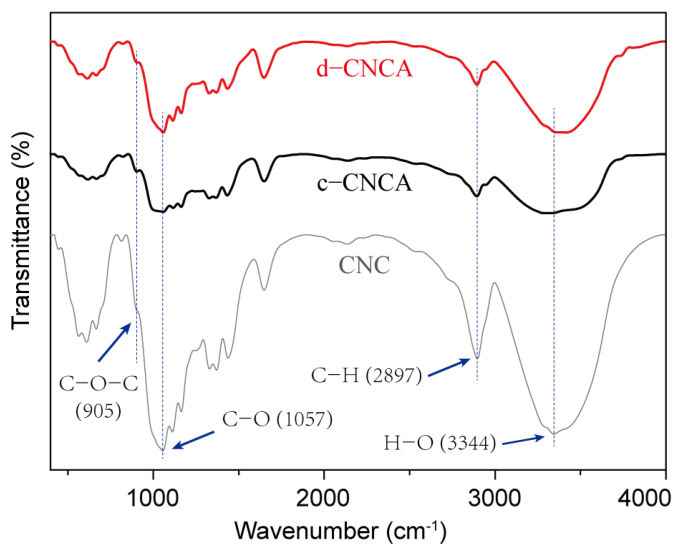
Comparison of Fourier-transform infrared (FTIR) spectra of c-CNCA, d-CNCA, and raw CNCs in the wavenumber region from 400 to 4000 cm^−1^.

The FTIR spectra of c-CNCA, d-CNCA, and raw CNCs in the range of 400–4000 cm^−1^ are compared in Figure 3. Similar to raw CNCs, both c-CNCA and d-CNCA present the typical characteristic peaks of cellulose molecules caused by C-O bond valence vibration (1057 cm^−1^), C-H bond stretching vibration (2897 cm^−1^), and H-O bond stretching vibration (3344 cm^−1^), respectively. This proves that the main components of CNCs were still well preserved after the dispersion and aggregation reactions [41]. Finally, the stretching vibration peak of *β*(1→4)-glycosidic bond (C-O-C) located at 905 cm^−1^ confirms that the main chains of cellulose are not destroyed during the chemical crosslinking process. Furthermore, c-CNCA and d-CNCA exhibit a distinct shrinkage of the peak at 3344 cm^−1^ compared to raw CNCs. The weakening of this absorption peak is mainly caused by the notable loss of inter- and intra-hydrogen bonds during the interaction between doped Ca^2+^ ions and the hydroxyl groups of cellulose [47]. Briefly, the addition of CaCl_2_ contributes to forming a stable cross-linked structure for the CNC aerogel.

### 2.3. Thermal Stability of CNC Aerogels

**Figure 4:**
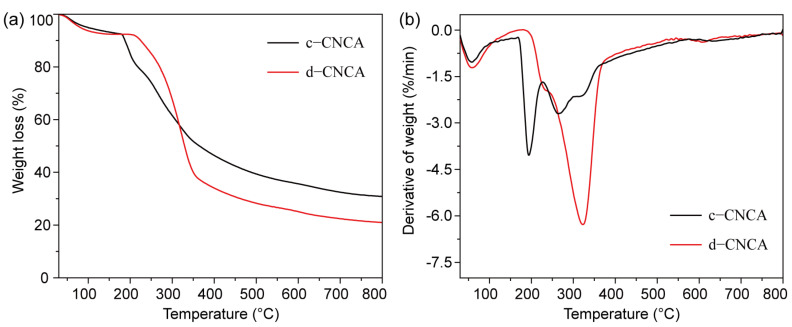
Thermogravimetric (**a**) and derivative thermogravimetry (**b**) curves of the fabricated CNC aerogels within the range from room temperature to 800 °C.

The thermal stability of c-CNCA and d-CNCA is reflected by their DG and DTG curves, as shown in Figure 4. The CNC aerogels exhibit a similar pyrolysis trend throughout the entire heating process (Figure 4a). In the initial pyrolysis stage (below 100 °C), the slight mass loss (around 5%) of the specimens mainly comes from the evaporation of free water captured from the air by the hydrogen bonds of the cellulose chains. As the temperature gradually increases, the pyrolysis of the sample enters the low-temperature stable stage (from 100 to 180 °C). At this stage, the relatively low temperatures are not sufficient to cause the pyrolysis of cellulose. Therefore, the minimal weight loss of c-CNCA (~3%) and d-CNCA (~2%) is mainly caused by the further evaporation of tiny amounts of residual water in the sample, generating flat TG curves. The considerable weight loss of these CNC aerogels occurs in the temperature range from 180 to 400 °C. During this period, the main body of the samples, i.e., cellulose, is severely decomposed by thermal oxidation [48], resulting in a significant decrease in sample weight (46% of c-CNCA and 58% of d-CNCA). When the temperature continues rising over 400 °C, the strong-bound water in each sample is basically lost, causing the TG curve to flatten gradually. Moreover, it can be seen from the DTG curve that the typical decomposition of d-CNCA occurs at a relatively high temperature (322.8 °C). This pyrolysis temperature is higher than that of c-CNCA (Figure 4b) as well as that of several reported cellulosic aerogels developed from Posidonia oceanica (below 300 °C) [16] and Kenaf core [20] biomass. The ordered porous structures of cellulose aerogels fabricated through unidirectional freezing demonstrated a higher thermal conductivity than disordered structures [30]. Hence, this higher decomposition temperature of d-CNCA may have resulted from its higher thermal conductivity, causing a better heat dissipation. Briefly, the good thermal stability (working temperature above 200 °C) of d-CNCA is sufficient to meet the application requirements under normal conditions.

### 2.4. Acoustic Absorption Performance of CNC Aerogels

**Figure 5:**
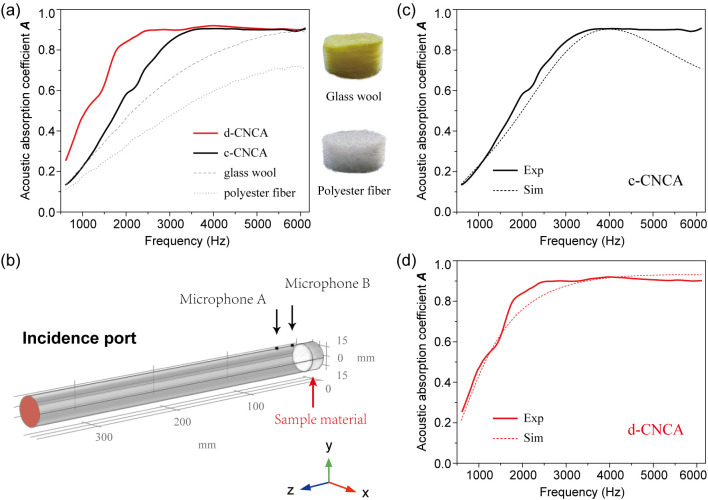
(**a**) Normal incidence acoustic absorption coefficient vs. frequency for c-CNCA, d-CNCA, and two types of typical commercial sound-absorbing material. (**b**) Schematic of the designed porous media model to predict acoustic absorption behavior. Comparison of the experimental (Exp, solid line) and simulated (Sim, dotted line) acoustic absorption coefficient for c-CNCA (**c**) and d-CNCA (**d**), respectively.

Figure 5 investigates the acoustic absorption behavior of the fabricated CNC aerogels, and their absorption properties are summarized in Table 2, wherein bandwidth means the frequency range with an absorption coefficient higher than 0.8. It can be clearly seen that the CNC aerogels exhibit a satisfactory ability to absorb audible sound even better than commercial glass wool and polyester fiber (Figure 5a). The ultra-low density of CNC aerogels plays a crucial role in achieving high impedance matching at the incident surface [49], allowing air molecules (e.g., O_2_ and N_2_) to easily enter the material’s interior without significant reflection of sound energy [50]. Additionally, the complex 3D interconnected porous structures of CNC aerogels are able to generate a considerable internal microstructured air–solid interfacial area. When airborne sound enters into the structure of the CNC aerogel, sound energy will be significantly dissipated at these interfacial areas through hybrid dissipation mechanisms including viscous loss, thermal loss, as well as a fairly long propagation path of sound waves caused by multiple reflections from the pore walls [51]. Hence, the high acoustic absorption of fabricated CNC aerogels can be briefly explained. Furthermore, it is noteworthy that the RCF and LnUF processes significantly impact the acoustic absorption properties of the CNC aerogels. As shown in Table 2, d-CNCA possesses a relatively higher acoustic absorption capacity than c-CNCA, especially in the lower frequency range from 600 to 3000 Hz (Figure 5a). Based on the above theory, the superior absorption performance of d-CNCA can also be interpreted as the rather advantageous physical properties caused by LnUF (e.g., lower density, higher porosity, etc.), leading to less surface reflection and more sufficient viscous/thermal dissipation of sound energy. Furthermore, its lower permeability would partly enhance the reflection of sound waves and prolong their propagation path in the material. Then, the longer path length of sound waves in d-CNCA may lead to a higher possibility of energy dissipation with a longer quarter-wavelength condition, thus enhancing absorption performance in the lower frequency region [52,53]. Finally, we designed a simple and feasible porous media model to verify the dissipation mechanism of sound energy in the fabricated CNC aerogels. For this purpose, Comsol Multiphysics was employed to develop a numerical simulation combining the finite element method with a three-parameter analysis JCAL model [54,55]. As shown in Figure 5b, the CNC aerogels were conceptualized as equivalent homogeneous media adhering to a rigid wall, and broadband sound waves were emitted and propagated within the tube and stroked the sample. Two microphones were used to detect sound pressure at different positions. Subsequently, the surface acoustic impedance of this uniform medium could be predicted by determining its three non-acoustic parameters (porosity, average aperture, and standard deviation of the average aperture *σ*) to further calculate the acoustic absorption coefficient. Here, the acoustic absorption coefficient of c-CNCA was simulated based on porosity and average aperture (Table 1) with a *σ* value of 0.54. The multi-scale aperture of c-CNCA (Figure 2e) endows it with good broadband acoustic absorption ability. Hence, a slight mismatch between theoretical and experimental results might happen at higher frequencies when the c-CNCA is regarded as a homogeneous medium in the simulation. Figure 2e also indicates that the pores with the most probable aperture occupy a considerable volume in d-CNCA, which means the most probable aperture can accurately reflect the pore size characteristics of the sample. Indeed, for d-CNCA, we obtained a simulated acoustic absorption curve that is very consistent with the experimental measurements (Figure 5d) by replacing the average aperture with the most probable aperture (standard deviation of 0.21). To a certain extent, the designed porous media model was able to predict the acoustic absorption behavior of the fabricated CNC aerogels and confirmed their dissipation mechanism for sound energy.

### 2.5. Multifunctional Properties of CNC Aerogels

**Figure 6:**
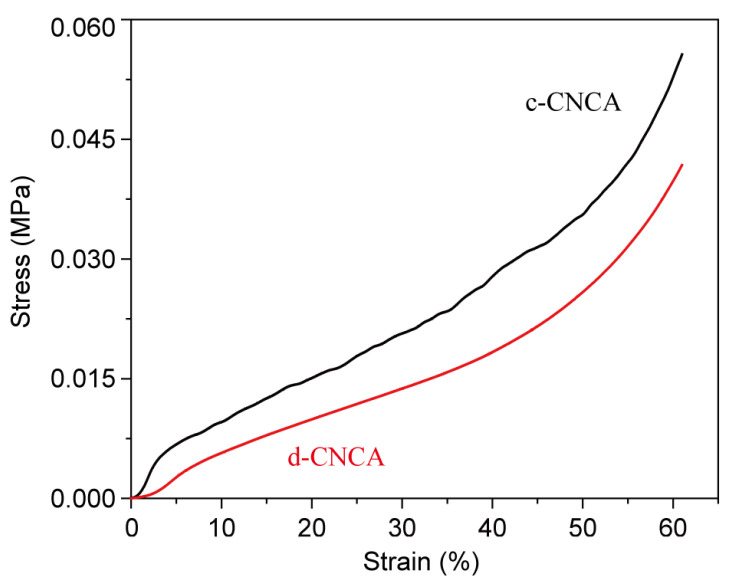
Stress–strain curves of c-CNCA and d-CNCA under compression loading.

In addition to assessing acoustic absorption performance, we simultaneously examined mechanical and optical properties to verify the multifunctionality of cellulose nanocrystal (CNC) aerogels. Mechanical properties were specifically evaluated through the axial quasi-static compression behavior of the samples. Briefly, the CNC aerogels contained a narrow elastic region quickly followed by non-linear deformation. This may have been due to the twisting or crumpling of pore walls during pressurization, thereby decreasing pore size and, in turn, pore volume. As strain continues to increase, the slope of the curve increases rapidly, indicating the densification of the CNC aerogels [18]. It has been proven that under the same freezing rate, the ordered pore structure formed by directional freezing possesses better anti-compression ability compared to the disordered structure obtained via conventional freezing [30,32]. However, compared with the LnUF process, the much slower freezing rate during RCF causes adequate growth of ice crystals with low nucleation and expansion speed, resulting in a denser structure in c-CNCA. Hence, the compression performance of the obtained CNC aerogels still shows a proportional relationship with bulk density. As shown in Figure 6, c-CNCA exhibited higher compressive resistance than d-CNCA throughout the entire deformation process, especially in terms of compressive Young’s modulus (40 kPa for c-CNCA and 10 kPa for d-CNCA). Remarkably, the Young’s modulus value for c-CNCA is higher than that of other reported sound-absorbing aerogels, e.g., nanofibrous aerogels (~21 kPa) [1], polyethylene terephthalate aerogels (1.16–2.87 kPa) [12], and pineapple fiber aerogels (1.64–5.73 kPa) [17], etc. This plays a pivotal role in ensuring mechanical stability when these aerogels are employed for practical applications.

**Figure 7:**
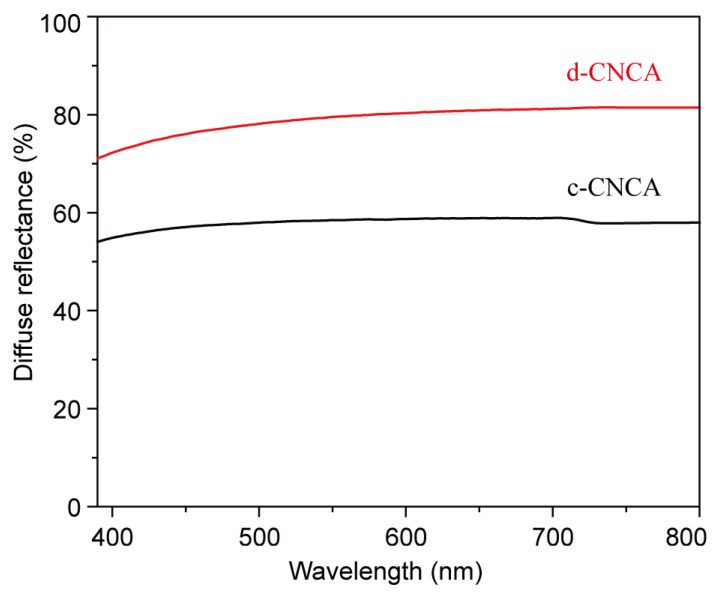
Diffuse reflection behavior of c-CNCA and d-CNCA in the visible light region.

As for optical performance, the effect of diffuse reflection in the visible range was investigated. Figure 7 shows diffuse reflectance as a function of wavelength for the fabricated CNC aerogels. It can be seen that both c-CNCA and d-CNCA exhibit a good ability to diffuse visible light, which is mainly attributed to the dense nanosized scattering centers on the surface giving them a white appearance [56,57]. Furthermore, d-CNCA (average reflectance of 79.16%) exhibits significantly higher broadband reflection compared to c-CNCA (average reflectance of 57.95%) across the visible light region. This phenomenon can be explained by the fact that light waves find it easier to pass through the large pores formed via the RCF process, resulting in a significantly higher transparency of light in c-CNCA, while for d-CNCA, the fine structure formed by the LnUF process efficiently reflects light waves, thereby exhibiting superior diffuse reflection performance. This outstanding optical property of the fabricated CNC aerogels may provide additional advantages in creating a bright and comfortable luminous environment when they are employed for indoor noise elimination.

## 3. Conclusions

We fabricated sustainable and multifunctional CNC aerogels through a simple and convenient dispersion/aggregation process followed by freeze-drying. The process of freeze-drying had an important influence on the porous structures of CNC aerogels, thereby significantly influencing their acoustic absorption performance. The slow freezing rate of the RCF process helped to form a denser structure composed of disordered, larger pores, and thus is suitable for rapidly fabricating an acoustic absorptive CNC aerogel with higher mechanical strength. Conversely, the liquid nitrogen unidirectional freezing (LnUF) process, with employs directional quick-freezing, yielded an ordered structure with lower density and smaller aperture. This resulted in higher porosity, improved thermal stability, superior diffuse reflectance in the visible light spectrum, and notably enhanced acoustic absorption capabilities, particularly in the low-frequency range, of the CNC aerogel. Future research will focus on improving the consistency between theoretical models and experiments to further optimize the acoustic absorption performance of CNC aerogels. The findings of this study offer valuable insights for tailoring the porous structures of aerogels, enabling the production of sound-absorbing materials with desired properties for a wide range of applications.

## 4. Materials and Methods

### 4.1. Materials

Cellulose nanocrystals (CNCs, crystallinity of 72%, length of 200 nm, and diameter of 10 nm) were provided by ScienceK New Material Technology Co., Ltd. (Suzhou, China). Calcium chloride anhydrous (CaCl_2_, ≥96%) was purchased from Xilong Scientific Co., Ltd. (Shantou, China). All chemical reagents were of analytical grade and were used as received without further purification. Deionized water (DI H_2_O) was of laboratory grade and was used throughout the experiment.

### 4.2. Fabrication of CNC Aerogels

In a typical synthesis (Figure 1), 1 g CNC was added and dispersed in 99 mL DI H_2_O by mechanically stirring for 1 h at room temperature. Then, the solution was placed in an ultrasonic cleaner (JP-100PLUS, Shenzhen, China) with a power of 1000 W to adequately disperse the CNC, resulting in a viscous transparent CNC dispersion. Next, 60 mL CaCl_2_ solution (0.05 mol/L) was added dropwise into above dispersion to cross-link the CNC and form a homogeneous CNC suspension. The CNC suspension was subsequently centrifuged via a high-speed centrifuge (JIDI-16D, Guangzhou, China) for 5 min at a speed of 10,000 rpm. After removing the supernatant, the residues were transferred to a set mold and densified to form a CNC hydrogel for freeze-drying. As shown in Figure 1, during the period of freeze-drying, the CNC hydrogel underwent two different freezing principles. For refrigerator conventional freezing (RCF), the CNC hydrogel was subjected to an omnidirectional cold source and frozen slowly at −20 °C for 24 h, while in the process of liquid nitrogen unidirectional freezing (LnUF), the CNC hydrogel was placed on top of an aluminum block, immersed in liquid nitrogen, and underwent ultra-low-temperature rapid freezing within 30 min. The unidirectional cold source formed a temperature gradient from bottom to top inside the CNC hydrogel. After completing the above freezing processes, the obtained frozen hydrogels were dried via a vacuum drier (NAI-L4-80D, Shanghai, China) at −80 °C under an ultra-low pressure of 1 Pa for 72 h to fabricate CNC aerogels. The CNC aerogel originating from the RCF process is named c-CNCA, and the one obtained from the LnUF process is named d-CNCA.

### 4.3. Characterization of CNC Aerogels

The macroscopic appearance of the CNC aerogels was observed through visual examination (i.e., optical photograph). Their microscopic morphology and structure were investigated using scanning electron microscopy (SEM, Hitachi S-3400 N, Tokyo, Japan) with an accelerating voltage of 15 kV. The samples were coated with an approx. 10 nm thick layer of platinum by sputtering to achieve clear imaging as a result of an improved conductivity of the sample surface. Further, the bulk density and porous characteristics (e.g., porosity, permeability, feature aperture, and pore size distribution pattern) of the CNC aerogels were quantitatively analyzed via a mercury porosimetry analyzer (Micromeritics AutoPore IV 9510, Atlanta, GA, USA) with a recognizable aperture ranging from 5 nm to 800 μm. A Fourier-transform infrared (FTIR) spectra spectrophotometer (Bruker Tensor 27, Saarbrucken, Germany) was employed to explore the chemical structures of the CNC aerogels in a wavenumber range from 400 to 4000 cm^−1^ with a resolution of 1.93 cm^−1^. The thermal stability of the CNC aerogels was assessed through thermogravimetric (TG) and derivative thermogravimetry (DTG) curves obtained by thermogravimetry (Netzsch TG 209 F1, Selb, Germany), and the samples were heated from room temperature to 800 °C at a heating rate of 10 K/min in nitrogen atmosphere. The acoustic absorption performance of the fabricated CNC aerogels, reflected by their normal acoustic absorption coefficient, was measured using a BSWA SW477 impedance tube (Beijing, China) based on a two-microphone transfer function method according to the ISO 10534-2:2023 standard [58]. The tested sound-absorbing frequency ranged from 600 to 6100 Hz. A uniaxial quasi-static compression test was performed to investigate the compressive performance (e.g., Young’s modulus) of the CNC aerogels. Samples were loaded into an MTS CMT6103 universal testing machine ( Eden Prairie, MN, USA) at a constant cross-head speed of 1 mm/min according to the GB/T 1041-2008 standard [59]. Samples used for acoustic and mechanical testing were processed in cylinders with a diameter of 30 mm and height of 16 mm. The diffuse reflectance of the specimens was measured through a spectrophotometer (Shimadzu UV-3600i Plus, Kyoto, Japan) covering visible light wavelengths from 390 to 800 nm.

## Figures and Tables

**Table 1:** Porous structural characteristics of c-CNCA and d-CNCA.

	Porosity (%)	Permeability (Darcy)	Average Aperture (μm)	Median Aperture (μm)	Most Probable Aperture (μm)
c-CNCA	92.36	207.50	179.30	262.27	292.23
d-CNCA	95.23	78.02	32.15	43.88	45.26

**Table 2:** Acoustic absorption properties of c-CNCA and d-CNCA.

	Maximum Absorption Coefficient (*A*_max_)	Average Absorption Coefficient (*A*_ave_)	Bandwidth (*A* > 0.8) (Hz)
c-CNCA	0.91 (at 4060 Hz)	0.72	3280
d-CNCA	0.92 (at 3978 Hz)	0.82	4320

## Data Availability

The raw/processed data required to reproduce these findings cannot be shared at this time as the data also form part of an ongoing study.
